# A directly negative interaction of miR-203 and ZEB2 modulates tumor stemness and chemotherapy resistance in nasopharyngeal carcinoma

**DOI:** 10.18632/oncotarget.11691

**Published:** 2016-08-30

**Authors:** Qingping Jiang, Ying Zhou, Huiling Yang, Libo Li, Xiaojie Deng, Chao Cheng, Yingying Xie, Xiaojun Luo, Weiyi Fang, Zhen Liu

**Affiliations:** ^1^ Department of Pathology, Third Affiliated Hospital of Guangzhou Medical University, Guangzhou 510150, China; ^2^ Cancer Research Institute, Southern Medical University, Guangzhou 510515, China; ^3^ Cancer Center, Traditional Chinese Medicine-Integrated Hospital, Southern Medical University, Guangzhou, Guangdong 510315, China; ^4^ Department of Pathology, Medical University of Guangzhou, Guangzhou 510182, China; ^5^ Sino-American Cancer Research Institute, Guangdong Medical College, Dongguan 523808, China

**Keywords:** miR-203a, ZEB2, NPC, tumor stemness, chemotherapy resistance

## Abstract

miR-203 is a tumor suppressor that is disregulated in numerous malignancies including nasopharyngeal carcinoma (NPC). However, the role of miR-203 in suppressing tumor stemness, chemotherapy resistance as well as its molecular mechanisms are unclear. In this study, we observed that miR-203 suppressed cell migration, invasion, tumor stemness, and chemotherapy resistance to cisplatin (DDP) *in vitro* and *in vivo*. miR-203 exerted these effects by targeting ZEB2 and downstream epithelial-mesenchymal transition (EMT) and tumor stemness signals. Interestingly we observed that miR-203 expression was directly suppressed by ZEB2 via targeting its promoter, which significantly reduced cell migration, invasion, tumor stemness, and chemotherapy resistance in NPC cells. Finally, we found that miR-203 was negatively correlated with ZEB2 expression in NPC tissues and tumor spheres. Our data demonstrate a directly negative feedback loop between miR-203 and ZEB2 participating in tumor stemness and chemotherapy resistance, highlighting the therapeutic potential of targeting this signal for NPC chemotherapy.

## INTRODUCTION

Nasopharyngeal carcinoma (NPC) is a type of malignant head and neck cancer derived from the nasopharyngeal epithelium, and one of the most common malignancies in Southern China and Southeast Asia. Many miRNAs have been documented to participate in NPC pathogenesis. Epstein-Barr virus-encoded microRNA nasopharyngeal carcinoma BART1 induces tumour metastasis by regulating PTEN-dependent pathways in NPC while miR-23 targets IL-8/Stat3 pathway sensitizing NPC cells to irradiation [1, 2]. miR-744 was observed to inhibit ARHGAP5 and induced NPC progression and metastasis in NPC [3]. miR-3188 is induced by Foxo1 through PI3K/AKT/c-Jun signaling which suppresses NPC cell growth via targeting mTOR [4]. miR-184 induced by PDCD4 directly inhibits c-Myc and Bcl-2, which ultimately suppresses NPC cell growth and induces apoptosis [5].

miR-203 disregulation contributes to the pathogenesis of many tumors through SNAI2 [6], CASK [7], and LASP1 [8]. In a recent investigation, miR-203 was shown to be negatively regulated and participate in LMP1-mediated induction of cell cycle transition. miR-203 has been found to directly target IL8/AKT signaling suppressing NPC radioresistance. Suppression for tumor stemness and chemotherapy resistance mediated by miR-203 has not been reported in the context of NPC.

ZEB2 encodes zinc finger E-box-binding homeobox 2 protein and is a member of the delta-EF1 (TCF8)/Zfh1 family of 2-handed zinc finger/homeodomain proteins. As an oncogenic transcription factor [9, 10], it has been reported to induce EMT and tumor stemness by regulating E-cadherin and miR-200 family members by binding to the promoter of these genes [11–13]. In previous studies, ZEB2 promoted NPC metastasis [14, 15], however the precise molecular mechanisms exerting this effect have not been determined.

In this study, we investigated the roles of miR-203 and ZEB2 on NPC cell migration, invasion, tumor stemness and chemotherapy resistance.

## RESULTS

### miR-203 overexpression suppresses cell migration and invasion *in vitro*

**Figure 1:**
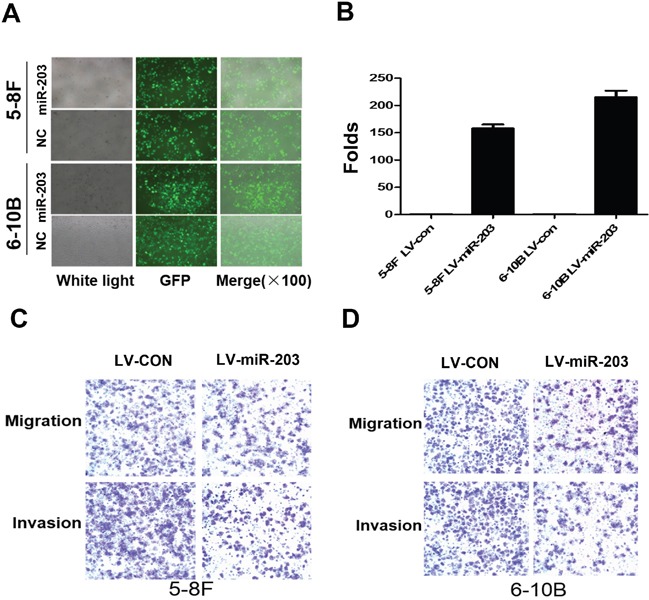
Overexpression of miR-203 inhibits NPC cell migration and invasion **A.** Lentivirus-mediated miR-203was infected into 5-8F and 6-10B cells. **B.** miR-203 was significantly upregulated after lentivirus-mediated miR-203 infection in 5-8F and 6-10B cells. **C.** Overexpression of miR-203 suppressed cell migration and invasion in 5-8F cells. **D.** Overexpression of miR-203 suppressed cell migration and invasion in 6-10B cells.

We established stable overexpression of miR-203 in 5-8F and 6-10B NPC cells using lentiviral infection (Figure 1A). Real time PCR confirmed that miR-203 was significantly increased in miR-203 overexpression NPC cells compared to control cells (Figure 1B). Transwell and Boyden assays revealed that miR-203 overexpression suppressed cell migration and invasion (Figure 1C, 1D).

### miR-203 overexpression reduces percentage of SP cells and tumor sphere growth

**Figure 2:**
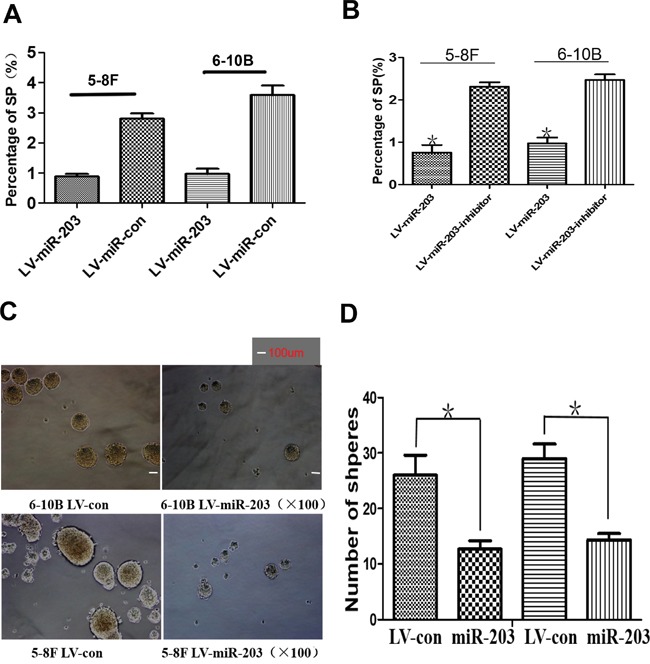
miR-203 overexpression reduces percentage of SP cells and tumor sphere growth **A.** Ectopic expression of miR-203 significantly suppressed the percentage of SP cells in 5-8F and 6-10B cultures. **B.** Specific inhibitor of miR-203 markedly restored the percentage of SP cells in miR-203-overexpressing 5-8F and 6-10B cells. **C.** Ectopic expression of miR-203 significantly reduced tumor sphere growth. **D.** Bar map indicated that miR-203 significantly decreased tumor sphere growth (*P<0.05)

The percentage of SP cells was markedly reduced in lines with miR-203 overexpression (Figure 2A). A miR-203 inhibitor significantly restored SP percentage comparable to levels in control NPC cells (Figure 2B). Furthermore, we observed that miR-203 overexpression decreased tumor sphere formation (Figure 2C, 2D).

### miR-203 reduces NPC cell resistance to DDP *in vitro* and *in vivo*

**Figure 3:**
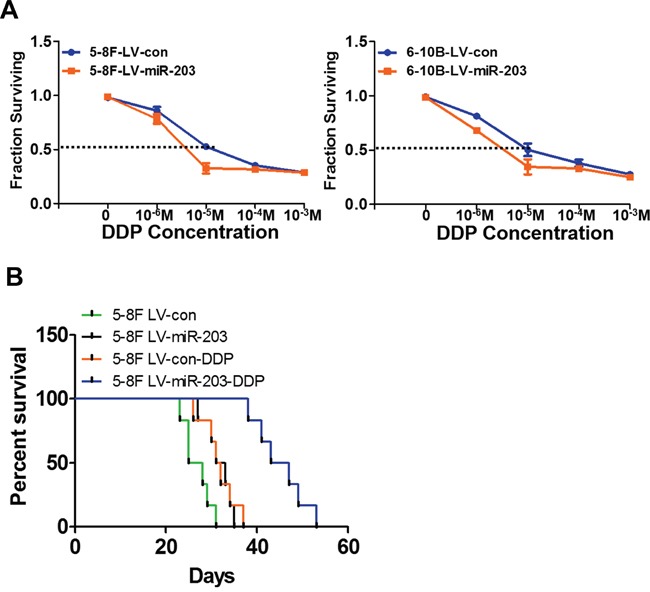
miR-203 significantly overcomes DDP resistance *in vitro* and *in vivo* **A.** Stable expression of miR-203 elevated NPC cell sensitivity to DDP. **B.** The Lv-con group treated with DDP and miR-203-overexpression group with NS injection had longer survival time than that of nude mice in Lv-con group with NS injection. However, nude mice in miR-203 overexpression group with DDP treatment had the longest survival time compared to the other three groups. (*P<0.05).

NPC cell lines with stable miR-203 overexpression exhibited significantly increased sensitivity to DDP. The IC50 to DDP was significantly reduced compared to control parental 5-8F (7.38±0.47 vs.13.48±0.62μm) and 6-10B cells (6.98±0.51 vs.12.98±0.71μm) (Figure 3A). Next we examined *in vivo* efficacy of DDP in mice bearing abdominal tumors originating from miR-203-overexpressing or control 5-8F cells. Using Kaplan-Meier analysis with the log-rank test, we found that the control group treated with DDP treatment and the untreated miR-203-overexpressing group had longer survival times than untreated control group mice. However, the miR-203-overexpressing group treated with DDP exhibited the longest survival time of the four groups (Figure 3B).

### miR-203 directly targets ZEB2 to modulate EMT and tumor stemness in NPC

**Figure 4:**
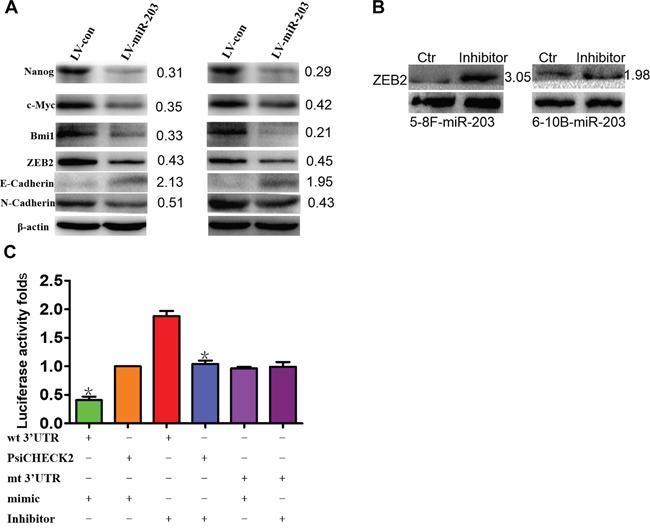
miR-203 modulates EMT and tumor stemness signals and directly targets ZEB2 **A.** Ectopic expression of miR-203 markedly modulated the expression of EMT and tumor stemness factors including suppressing ZEB2, N-cadherin, NANOG, and c-MYC, and BMI1 and induced E-cadherin expression in 5-8F and 6-10B cells. **B.** Inhibitor of miR-203 restored the expression of ZEB2. **C.** Luciferase reporter assay demonstrating that ZEB2 is a direct target of miR-203 in NPC (*P<0.05).

We observed that miR-203 overexpression modulated expression of EMT and tumor stemness factors including suppression of ZEB2, N-cadherin, NANOG, c-MYC, and BMI1 as well as induced E-cadherin expression (Figure 4A). We found that a miR-203 inhibitor significantly induced ZEB2 expression when introduced into miR-203-overexpressing 5-8F and 6-10B cells (Figure 4B). Next, wild-type (wt) or mutant (mt) ZEB2 3′UTR vectors were cotransfected with either miR-203 mimics or inhibitor into 5-8F cells. Luciferase activity of wt vector was significantly reduced by miR-203 mimics (Figure 6B, lane 1; P<0.001) but was notably increased by a miR-203 inhibitor (Figure 6B, lanes 3; P=0.008). The activity of mt vector was unaffected (Figure 4C, lanes 5 and 6) by either miR-203 mimics or inhibitor cotransfection. Taken together, these results strongly supported that ZEB2 is a direct target of miR-203 in NPC cells.

### The interfering efficiency of siZEB2s in NPC cells

By real-time PCR, siZEB2-1 exhibited the best knockdown efficiency in 5-8F (79%) and SUNE1 (76%) cells (Supplementary Figure S1A). Immunofluorescence (Supplementary Figure S1B) and western blot (Supplementary Figure S1C) confirmed significant ZEB2 downregulation mediated by siZEB2-1 in 5-8F and SUNE1 cells

### Knocking down ZEB2 suppresses cell migration and invasion by regulating EMT signals

**Figure 5:**
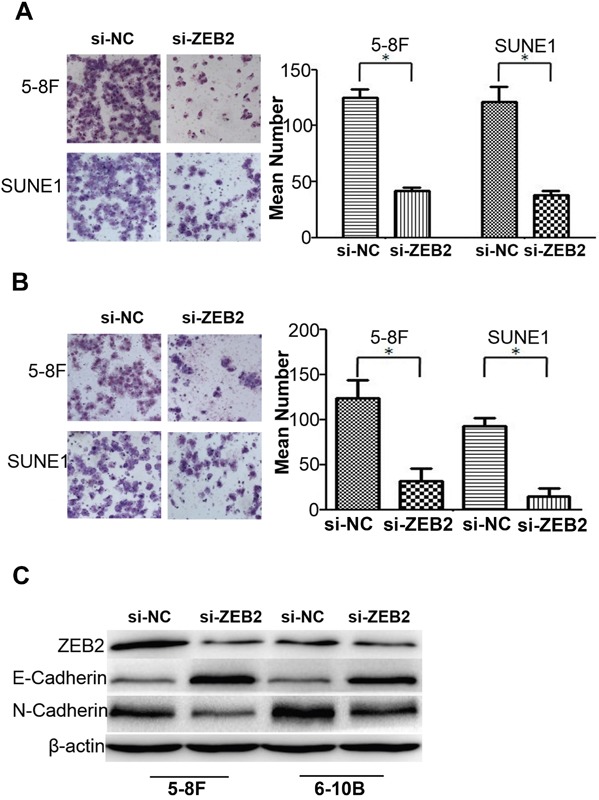
Knocking dwon ZEB2 suppresses cell migration and invasion by regulating EMT signals in NPC **A** and **B.** Knocking dwon ZEB2 suppresses cell migration and invasion in NPC 5-8F and 6-10B by transwell and boyden assays. **C.** Suppression of ZEB2 significantly stimulated the expression of E-cadherin and reduced N-cadherin expression in NPC cells (*P<0.05).

Cell migration (Figure 5A) and invasion (Figure 5B) were dramatically reduced after knocking down ZEB2 in 5-8F and SUNE1 cells compared to their respective controls. Further, we observed that suppression of ZEB2 significantly stimulated expression of E-cadherin and reduced N-cadherin (Figure 5C).

### Suppression of ZEB2 reduces SP cells and chemotherapy resistance by modulating tumor stemness signals

**Figure 6:**
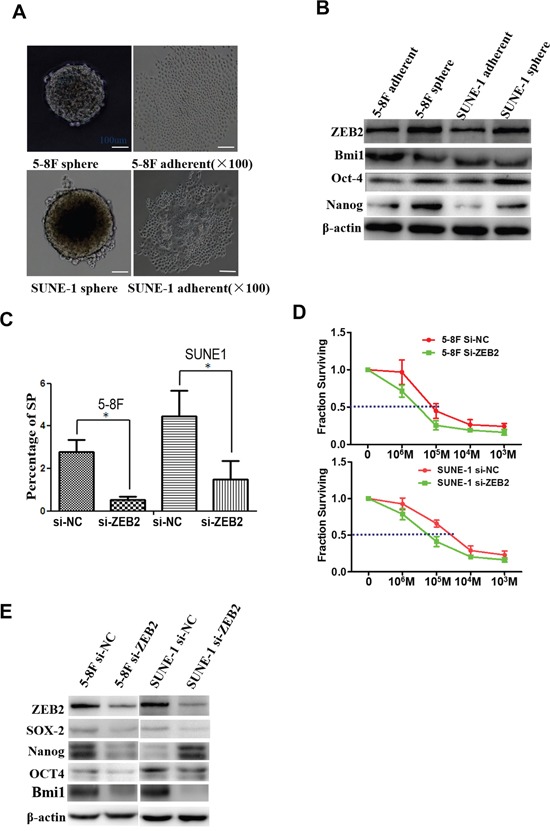
Suppression of ZEB2 reduced SP cells and chemotherapy resistance to DDP by modulating tumor stemness signal in NPC **A.** Tumor spheres derived from 5-8F and SUNE1 cells. **B.** Protein levels of ZEB2, BMI1, NANOG, and OCT4 were markedly decreased in tumor spheres compared to cells grown in standard 2D cultures. **C.** Knocking down ZEB2 significantly reduced the percentage of SP cells in 5-8F and SUNE1 cultures. **D.** Repression of ZEB2 induced chemotherapy sensitivity. **E.** ZEB2 suppression significantly reduced the expression of SOX2, NANOG, OCT4, and BMI1 in 5-8F and SUNE1 cells (*P<0.05).

We next examined ZEB2 expression as well as other tumor stemness factors in tumor spheres compared to standard culture conditions (Figure 6A). Protein levels of ZEB2, BMI1, OCT4, and NANOG were decreased in tumor spheres compared to parental NPC cells (Figure 6B). Further, the percentage of SP cells was significantly reduced after knocking down ZEB2 by siRNA (Figure 6C). NPC cell sensitivity to DDP was also markedly increased after ZEB2 downregulation (5-8F:6.85±0.66 vs.12.57±0.39; SUNE1:9.12±0.33 vs.37.25±1.17) (Figure 6D). Finally, we observed that ZEB2 suppression significantly reduced the expression of SOX2, NANOG, OCT4, and BMI1 in NPC cells (Figure 6E).

### ZEB2 directly suppresses the expression of miR-203 by binding its promoter

**Figure 7:**
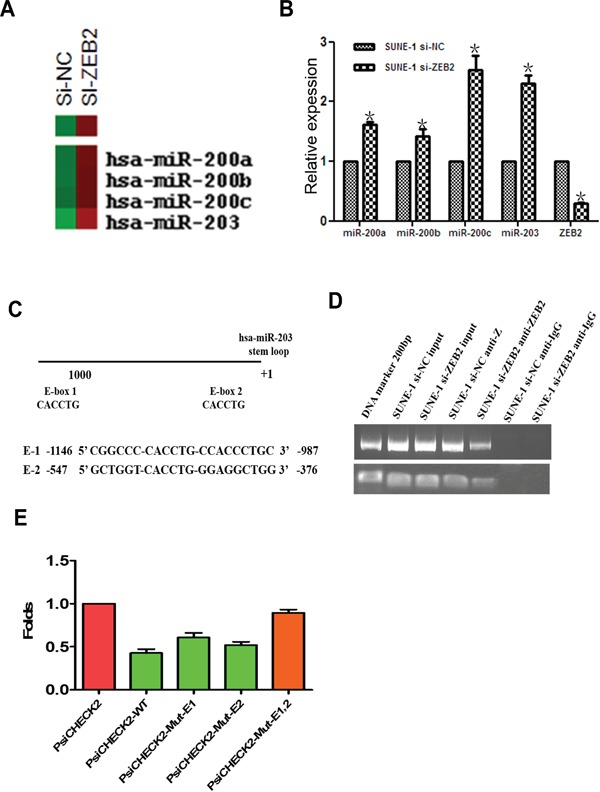
ZEB2 directly suppresses miR-203 expression by binding its promoter **A.** miRNA array indicated that miR-203 and miR-200 family members were upregulated after knockdown of ZEB2 in SUNE1 cells. **B.** Real-time PCR confirmed increased expression of miR-203 and miR-200 family members. **C.** The predicted E-box binding site of ZEB2 in the miR-203 promoter. **D.** DNA ChIP assay displayed that ZEB2 directly binds to the miR-203 promoter (E1 and E2) (*P<0.05). **E.** Luciferase reporter assay indicated that ZEB2 suppressed the activity of miR-203 promoter.

To investigate molecular mechanism of ZEB2, we used Affymetrix 3.0 miRNA array to examine the differential expression profile of ZEB2-suppressed SUNE1 cells. The results indicated that expression of miR-203, miR-200a, miR-200b, and miR-200c were upregulated after ZEB2 knockdown (Figure 7A). Further, Real-time PCR confirmed the reliability of these data (Figure 7B). Further, we observed that ZEB2 could directly bind to the miR-203 promoter in E1 and E2 boxes (Figure 7C, 7D). Furthermore, an increase of luciferase activity in the wildtype miR-203 promoter, sites E1 mutation alone, or E2 mutation alone was observed upon downregulation of ZEB2 in SUNE1 cell lines (P<0.05) compared to the empty vector and miR-203 promoter with the mutation of site E1 and E2 (Figure 7E).

### ZEB2 antagonized miR-203 increasing cell migration, invasion, stemness, and drug-resistance

**Figure 8:**
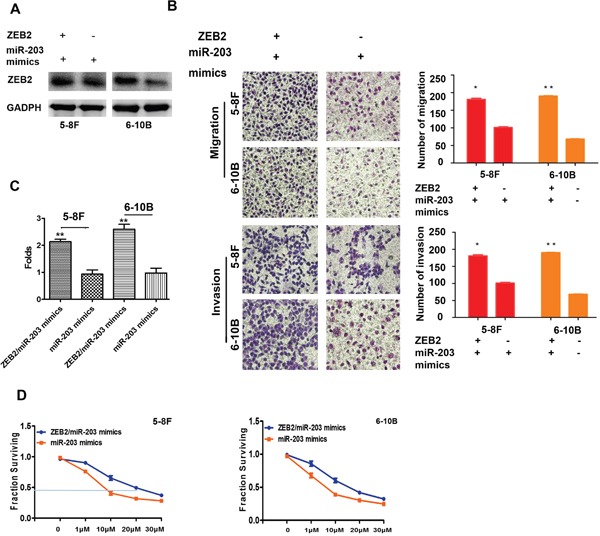
ZEB2 antagonized miR-203 increasing cell migration, invasion, stemness, and drug-resistance **A** and **B.** Cell migration and invasion were markedly increased in NPC cells with the transfection of miR-203 mimics and ZEB2 cDNA compared to cells with miR-203 mimics. **C.** The percentage of SP cells was elevated in NPC cells with the transfection of miR-203 mimics and ZEB2 cDNA compared to that of miR-203 mimics transfection. **D.** ZEB2 significantly elevated the DDP-resistance in NPC cells with the transfection of miR-203 mimics and ZEB2 cDNA compared to that of miR-203 mimics transfection.(*P<0.05,**P<0.01).

We observed that cell migration (Figure 8A), invasion (Figure 8B), and the percentage of SP cells (Figure 8C) were dramatically increased in cells with the transfection of miR-203 mimics and ZEB2 cDNA compared to cells with miR-203 mimics in NPC. Furthermore, we also found that ZEB2 significantly elevated the DDP-resistance in NPC cells with the transfection of miR-203 mimics and ZEB2 cDNA compared to that of miR-203 mimics transfection (5-8F:20.38±1.31 vs.8.72±0.66; 6-10B:17.01±1.82 vs.7.45±0.57) (Figure 8D).

### miR-203 expression is negatively correlated with ZEB2 in NPC tumor spheres and NPC tissues

**Figure 9:**
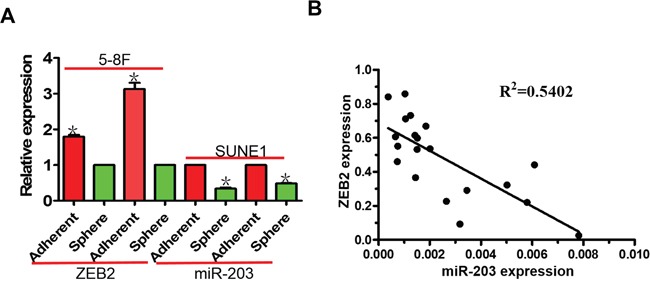
miR-203 is negatively correlated with ZEB2 expression in NPC tumor spheres and NPC tissues **A.** miR-203 expression was downregulated in NPC tumor spheres compared to cells grown in standard 2D cultures. Inversely, ZEB2 mRNA expression was upregulated in NPC tumor spheres. **B.** miR-203 expression negatively correlated with ZEB2 in NPC tissues (*P<0.05).

In NPC tumor spheres, we observed decreased miR-203 expression and increased ZEB2 expression compared to their parental NPC cells (Figure 9A). In addition, we observed that miR-203 was negatively correlated with ZEB2 mRNA level in NPC tissues (Figure 9B).

## DISCUSSION

miR-203 is located in chromosome 14q32 and displays a highly organ- and tissue-specific expression pattern across 21 human organs and tissues analyzed [16]. Recently more studies have demonstrated that miR-203 is a key tumor suppressor participating in the pathogenesis of many tumors including nasopharyngeal carcinoma. Yu et al. observed that miR-203 was negatively modulated by LMP1 causing direct targeting of E2F3 and CCNG1 which contributed to NPC cell cycle transition [17]. Further, miR-203 was observed to reduce nasopharyngeal carcinoma radioresistance via IL-8/AKT signaling [18]. However, the role of miR-203 in modulating cell migration, invasion, tumor stemness, and chemotherapy resistance as well as its detailed molecular mechanisms have not been reported in NPC.

In this investigation, we established stable miR-203-overexpressing NPC cell lines and observed cell migration and invasion compared to their respective parental control cells. This result was similar to other reports in the contexts of prostate cancer, esophageal squamous cell carcinoma, and breast cancer [19–22]. Further, we found that miR-203 significantly suppressed NPC tumor stemness features including reducing SP cells percentage and tumor sphere growth. This finding was similar to Wu, Ju, and Taube's findings [23–25]. Finally, miR-203 was observed to significantly reduce NPC cell resistance to DDP *in vitro* and markedly prolong the survival time of nude mice bearing abdominal tumor. Consistent with Ru's finding in breast cancer [26], miR-203 can significantly reduce the potential for chemotherapy resistance. It is well known that EMT and tumor stemness signals participate in chemotherapy resistance [27–29] We confirmed that miR-203 suppressed cell migration, invasion, tumor stemness, and DDP resistance by blocking EMT and stemness signal factors. This included downregulation of ZEB2, N-cadherin, BMI1, c-MYC, and NANOG, while inducing E-cadherin. Interestingly, ZEB2 is an EMT and tumor stemness co-inducer and thus has been confirmed as a direct target of miR-203 in NPC cells.

ZEB2 is a key factor which promotes metastasis in some tumor types [16, 17, 30]. We observed that knocking down ZEB2 significantly suppressed cell migration and invasion by inhibiting the EMT pathway via induction of E-cadherin and suppression of N-cadherin. This further supported the role of ZEB2 in inducing EMT [14, 31]. In previous studies, ZEB2 promoted stem cell properties in prostate, head and neck, and ovarian cancers [32–34]. However, there have not been reports demonstrating ZEB2's role in inducing tumor stemness. We observed increased ZEB2 expression in tumor spheres compared to parental NPC cells. Knocking down ZEB2 significantly suppressed the percentage of SP cells and DDP resistance by reducing the expression of tumor stemness factors including BMI1, SOX2, NANOG, and OCT4. In prvious documents, BMI1 and SOX2 were the key tumor stemness factors [35–39], which had been documented as the direct targets of miR-200 family members. Interestingly, ZEB2 was the directly upstream regulator of miR-200 family members. Thus, We guessed that ZEB2 induced tumor stemness through modulating miR-200/BMI1/SOX2 signals in NPC. Together the above-mentioned data indicate the effect of ZEB2 suppression was consistent with miR-203 function, demostrating ZEB2 as a key oncongene that participates in NPC pathogenesis.

To explore the molecular mechanisms of ZEB2 in NPC, Affymetrix miRNA array and real-time PCR were used to examine differential miRNA expression. In addition to miR-200 family members (miR-200a, b, and c) which are known ZEB2 targets, miR-203 was also found to be negatively modulated by ZEB2. We confirmed that ZEB2 could directly bind to the miR-203 promoter by E-boxes. Furthermore, we observed that ZEB2 directly antagonized miR-203 increasing cell migration, invasion, stemness, and drug-resistance. Finally, Expression of miR-203 was negatively correlated with ZEB2 in NPC tissues and cultured tumor spheres. These results demonstrate that miR-203 is negatively modulated by ZEB2 in NPC.

Taken together, our studies suggest a negative feedback loop between ZEB2 and miR-203 which promotes NPC pathogenesis by inducing tumor stemness and chemotherapy resistance.

## MATERIALS AND METHODS

### Sample collection and cell culture

NPC cell lines 5-8F, 6-10B, and SUNE1 were obtained from the Cancer Research Institute of Southern Medical University and maintained in RPMI 1640 medium supplemented with 10% Fetal Bovine Serum (FBS) (PAA Laboratories, Inc, Pasching, Austria) in a humidified chamber with 5% CO_2_ at 37°C. Twenty (20) fresh NPC were obtained from an otorhinolaryngologist using a nasal endoscope. All samples obtained were immediately flash frozen in liquid nitrogen. Clinical processes were approved by the Ethics Committees of People's Hospital of Zhongshan City and patients gave informed written consent.

### RNA isolation and qRT-PCR

RNA was extracted from NPC cell lines, NPC tissues and normal nasopharynx tissues using Trizol (Takara, Shiga, Japan). For miR-203 qRT-PCR expression analysis, mature miRNAs were reverse-transcribed, and real-time PCR was performed using All-in-One™ miRNA qRT-PCR Detection Kit following the manufacturer's protocol. (GeneCopoeia™, Cat.No: AOMD-Q020). For ZEB2 qRT-PCR, RNA was transcribed into cDNA and amplified with specific sense/antisense primer [40]. The assays were performed in accordance with manufacturer's instructions (Takara, Shiga, Japan). PCR reactions for each gene was repeated three times. miRNA and mRNA expression was normalized to U6 and ARF5, respectively using the 2-ΔΔCt method as previously described [4].

### Construction of lentivirus-mediated miR-203 overexpression in NPC cells

Lentivirus (GV209) particles carrying hsa-pri-miR-203 precursor or its control were prepared and lentiviral transduction of 5-8F and 6-10B cells was performed according to the manufacturer's protocol (Shanghai Genechem Co., Ltd). The resulting cells were seeded onto 96-well plates and cultured for 3 weeks to produce stable miR-203-overexpressing 5-8F and 6-10B cells. Ectopic expression of miR-203 was confirmed by quantitative RT-PCR.

### Cell migration and invasion

Cell migration and invasion assays were carried out according to a previous description [41]. All assays were independently repeated three times. Cell invasion assay protocol was similar to the cell migration assay except transwell membranes were precoated with 24 μg/μl Matrigel (R&D Systems, USA).

### Side-population cells

5-8F, 6-10B, and SUNE1 NPC cells were cultured for 24 hrs, and then NPC cells were washed three times with PBS. Further, these cells were digested with 0.25% trypsin, washed twice with calcium/magnesium-free PBS, resuspended in ice-cold RPMI-1640 medium (supplemented with 2% FBS) at a concentration of 1×10^6^ cells/mL, and incubated at 37°C in a 5% CO_2_ incubator for 90 min. Following this, the changes in the percentage of SP cells were analyzed by flow cytometry (BD FACSAria), as previously described [23].

### Tumor sphere formation

After culturing 5-8F, 6-10B, and SUNE1 NPC cells for 24 hrs, these cells were washed three times with PBS. Subsequently, 9000 cells were plated in 6-well ultra low attachment plates (Corning, Corning, NY) in serum-free DMEM-F12 (Hyclone), supplemented with 20 ng/mL epidermal growth factor (Peprotech), 10 ng/mL basic fibroblast growth factor (Peprotech), and B27 supplement (1:50 dilution; BD). After 7 days of culture, tumor spheres were counted under an inverted microscope.

### Western blot

Western blot was performed based on previous descriptions [12, 13] with rabbit polyclonal ZEB2 and GAPDH (Santa Cruz Biotechnology, USA); c-MYC, BMI1, SOX2, OCT4, NANOG, E-cadherin, N-cadherin antibody (Cell Signaling Technology, Danvers, USA). An HRP-conjugated anti-rabbit IgG antibody was used as the secondary antibody (Zhongshan, Beijing, China). Signals were examined using enhanced chemiluminescence reagents (Pierce, Rockford, IL) and a Bio-RAD ChemiDox XRS.

### Knockdown efficiency of siZEB2s in NPC cells

Three siRNAs (Supplementary Table S1) was purchased by Riobo Corporation, Guangzhou City China. Knockdown efficiency of siZEB2s was examined by real-time PCR, immunofluorescence, and western blot.

### miRNA array for siZEB2

Affymetrix miRNA array 3.0 was used to examine the differential miRNA expression in siZEB2 and siCtr 5-8F cells. Experiment was performed and analyzed at Gene Corporation, Shanghai, China.

### Chromatin immunoprecipitation assay

Chromatin immunoprecipitation assay was carried out according to previous descriptions [4, 5]. DNA–protein complexes were immunoprecipitated from SUNE1 cells after ZEB2 cDNA transfection using the Chromatin Immunoprecipitation Kit (Millipore, Billerica, MA, USA), according to the manufacturer's protocol with 1 mg polyclonal ZEB2 antibody or 1mg normal mouse IgG (Millipore). Precipitated DNA was subjected to qPCR analysis using spcific primers (−547-Forward: 5′GCTGGTCCTCACCTGTTCC3′;-547-Reverse: 5′CCAGCCTCCAGCGCC3′; -1146-Forward:5′CGGCCCATGTGGAAATGTCT3′, -1146-Reverse:5′GCAGGGTGGTGACCATTCAT3′) to amplify across the miR-203 promoter region [14]. Data was analyzed using the 2^−ΔΔCt^ method.

### Luciferase reporter assay

To generate a miR-203 promoter vector, a chemistry synthesized 1291-bp fragment containing the 2 binding sites of ZEB2 was inserted into a psiCHECK-2 luciferase reporter vector. In addition, ZEB2-binding site mutation vectors were constructed according to the instruction of site-directed mutagenesis kit. These psiCHECK-2-derived vector and siZEB2 were cotransfected into NPC SUNE1 cells using Lipofectamine 2000 Reagent (Invitrogen).

### Transient transfection with miR-203 inhibitor, si-ZEB2, or ZEB2 cDNA

miR-203 inhibitor and si-ZEB2s were purchased from RiboBio Inc, Guangzhou, China. ZEB2 cDNA was chemically synthesized and then constructed into adenovirus vector (Shanghai Genechem Co., Ltd., China). Twenty-four hours before transfection, NPC cells were plated onto a 6-well plate (Nest, Biotech, China) at 30–50% confluence. They were then transfected into cells using TurboFectTM siRNA Transfection Reagent (Fermentas, Vilnius, Lithuania) according to the manufacturer's protocol. Cells were collected after 48-72 hrs for further experiments.

### miRNA target validation

ZEB2 was predicted to be directly regulated targets of miR-203 based on miRanda software analysis (University of Heidelberg, Mannheim, Germany). A 295 fragment of the ZEB2 3′UTR was amplified by PCR and cloned into psiCHECK-2 vectors and is referred to as wt. Site-directed mutagenesis of the miR-203 binding site in ZEB2 3′UTR was performed using GeneTailor Site-Directed Mutagenesis System (Invitrogen) and is referred to as mt. For reporter assays, wt or mt vector and the control vector psiCHECK-2 vector were cotransfected into SUNE1 cells with miR-203 mimics or inhibitor. Luciferase activity was measured 48 hrs after transfection using the Dual-Luciferase Reporter Assay System (Promega Corporation, Madison, WI, USA).

### MTT cytotoxicity assay

NPC 5-8F and 6-10B cells with miR-203 overexpression or ZEB2 suppression were seeded in 96-well plates at 5 × 10^3^ cells/well. Once cells attached, they were treated with DDP at 1, 10, 100, or 1000 μM (0.5mg/ml) and incubated at 37°C in 5% CO_2_ for 48 hrs. Subsequently, 10 μl of MTT (5 mg/ml) (Sigma, StLouis, MO, USA) was added to each well, and the plates were incubated at 37°C for 4 hrs. Supernatants were removed and 100 μl of DMSO (Sigma) was added to each well. The absorbance value (OD) of each well was measured at 490 nm and half maximal inhibitory concentration (IC50) was calculated. Experiments were performed three times.

### Experiments for DDP Treatment in nude mice

*In vivo* experiments were approved by the Animal Care and Use Committee of Southern Medical University. Forty (40) 4-week nude mice (BALB/C, nu/nu) weighing 12-13g were provided by the Central Animal Facility of Southern Medical University. To establish an NPC mouse model, 6 × 10^5^ miR-203-overexpressing 5-8F cells (N=20) or control cells (N=20) were intraperitoneal injected in 0.2 mL buffered saline into nude mice. Tumors were allowed to grow for 3 days and then the animals were divided into four groups: control cell group (NC)+Normal saline (NS), miR-203+NS, NC+DDP and miR-203+DDP (each group: N=10). Mice treated with miR-203-overexpressing 5-8F cells and their controls were intraperitoneal injected with NS or DDP every three days, respectively. The weight and survival of nude mice was measured regularly.

### Statistical analysis

All data were analyzed for statistical significance using SPSS 13.0 software. Student's t-test was applied to examine differences in mRNA expression levels of ZEB2 and miR-203. The relationship between ZEB2 and miR-203 levels was analyzed usingSpearman test. Two-tailed Student's t-test was used for comparisons of two independent groups. One-way ANOVA was used to determine the differences between groups for all *in vitro* analyses. A P value of less than 0.05 was considered statistically significant.

## SUPPLEMENTARY MATERIAL FIGURE AND TABLE


