# Supplementary material for: A directly negative interaction of miR-203 and ZEB2 modulates tumor stemness and chemotherapy resistance in nasopharyngeal carcinoma
Source: Oncotarget. 2016 Aug 30;7(41):67288–301. doi: 10.18632/oncotarget.11691 (PMC5341875; doi:10.18632/oncotarget.11691)
Supplement: Supplementary file 1 [file oncotarget-07-67288-s001.pdf]

A directly negative interaction of miR-203 and ZEB2 modulates tumor stemness and chemotherapy resistance in nasopharyngeal carcinoma

SUPPLEMENTARY FIGURE AND TABLE

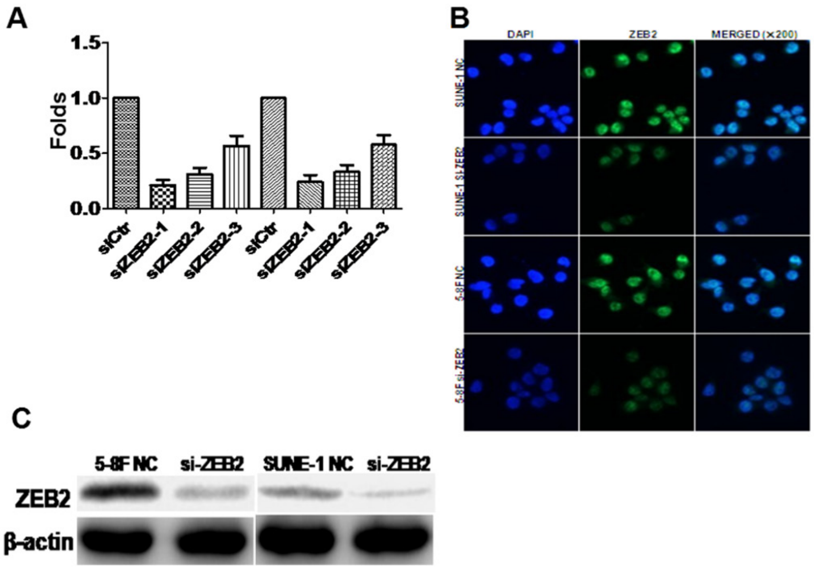

Supplementary Figure S1: Knockdown efficiency of siZEB2s in NPC cells. A. Real-time PCR demonstrated that siZEB2-1 exhibited best interfering efficiency in 5-8F (79%) and SUNE1 (76%) cells. **B** and **C.** Immunofluorescence and western blot confirmed significantly downregualated ZEB2 induced by siZEB2-1 in 5-8F and SUNE1 cells.

Supplementary Table S1: The sequences of siZEB2

Si-ZEB2 sequence	sense	anti-sense
siRNA-001	5'- GGACACAGGUUCUGAAACA dTdT-3'	3'- dTdT CCUGUGUCCAAGACUUUGU-5'
siRNA-002	5'- CUGCAAGGCUGAAGAAAUU dTdT-3'	3'- dTdT GACGUUCCGACUUCUUUAA-5'
siRNA-003	5'- CAAAUAAUCUGGACAACAA dTdT-3'	3'- dTdT GUUUAUUAGACCUGUUGUU-5'
